# T59, a New Compound Reconstructed from Curcumin, Induces Cell Apoptosis through Reactive Oxygen Species Activation in Human Lung Cancer Cells

**DOI:** 10.3390/molecules23061251

**Published:** 2018-05-24

**Authors:** Zhendong Zhao, Yanjun Yang, Weihai Liu, Ziqian Li

**Affiliations:** 1 School of Chinese Materia Medica, Guangdong Food and Drug Vocational College, No. 321 Longdong bei Road, Guangzhou 510520, China; zhaozd2008@126.com (Z.Z.); ncyyj@163.com (Y.Y.); liuweihai@outlook.com (W.L.); 2 Department of Microbial and Biochemical Pharmacy, School of Pharmaceutical Sciences, Sun Yat-sen University, Guangzhou 510006, China

**Keywords:** T59, A549, H1975, cell apoptosis, ROS, PI3K/AKT

## Abstract

Curcumin is acknowledged for its antioxidant, anti-inflammatory, anti-cancer, and wound-healing properties. However, the biological activity and the molecular mechanisms of T59, which is a new derivative of curcumin, are not fully understood. The present study was aimed to determine the cytoxicity role of T59 in human lung cancer and the molecular mechanisms. Cytotoxicity and cell apoptosis effects induced by T59 were determined by MTT, AO staining, Annexin V, and JC-1. Compared with curcumin, T59 exerted more effective cytotoxicity and cell apoptosis effects in A549 and H1975. With the decreasing level of the mitochondrion membrane potential, the generation of reactive oxygen species (ROS) was increased and induced by T59. Furthermore, the expressions of cleaved-caspase-3 and Bax were increased, which were reversed by NAC mainly through the PI3K/AKT signaling pathway. Our results suggested that T59 has the potential for further investigation and study to act as an anti-cancer therapeutic against human lung cancer.

## 1. Introduction

Lung cancer, which is one of the leading causes of cancer death around the world [1], shows an increasing level of reactive oxygen species (ROS) than normal cells [2,3]. However, with the increasing level of ROS, cells will be subject to irreversible oxidative damage [4,5]. Therefore, more and more studies on the destruction of the maintenance of redox balance have been raised [6,7,8].

**Figure 1:**
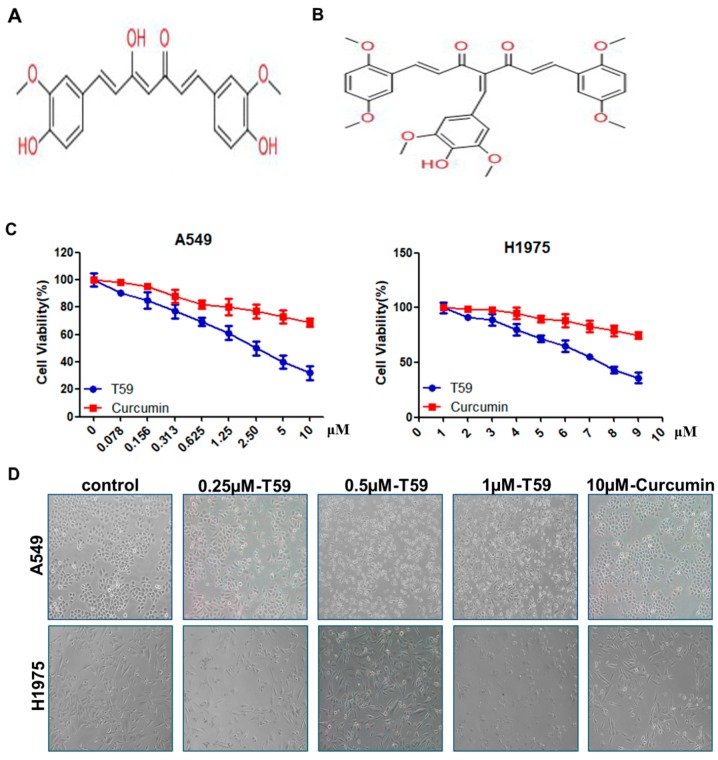
Structure of T59 and the cytotoxicity of T59. (**A**) Structure of curcumin [C_21_H_20_O_6_, 1,7-bis-(4-hydroxy-3-methoxyphenyl)-hepta-1,6-diene-3,5-dione]. (**B**) Structure of curcumin derivative T59 [C_32_H_32_O_9_]. (**C**) Cytotoxicity of T59 and curcumin on human lung cancer cells A549 and H1975 were measured by MTT assay at 24 h. (**D**) Representative images (×100) of cellular morphology after treated with 0.250, 0.500, and 1.00 μM T59 and with 10.000 μM curcumin for 24 h.

Curcumin, [1,7-bis-(4-hydroxy-3-methoxyphenyl)-hepta-1,6-diene-3,5-dione] (see Figure 1A), is an active polyphenolic compound from *Curcuma longa* L. with antioxidant [9], anti-inflammatory [10], and wound-healing properties [11]. Emerging evidence indicates that curcumin and its chemical analogs increased intracellular ROS and mediated the anti-cancer effects in various cancers [12,13,14]. However, the bioavailability of curcumin was low and the solubility was poor [15]. As a result, the application of curcumin was limited. Therefore, many derivatives from curcumin were discovered recently [16]. T59, which is a novel derivative of curcumin (see Figure 1B), was synthesized and kindly donated by Professor Bu [17]. This may have higher bioavailability than curcumin. In the present study, we aimed to analyze the bioactivity of T59 and its molecular mechanisms. We compared the effects of T59 with curcumin on cytoxicity activity, and we are the first to show that T59 is more effective than curcumin in suppressing cell proliferation and induction apoptosis in human lung cancer cells (A549 and H1975), together with AKT phosphorylation. Therefore, we suggest that T59 is substantially more effective than curcumin in vitro.

## 2. Results

### 2.1. Effects of T59 on Cytotoxicity

In comparison with the curcumin, T59, which is a reconstructed form of curcumin, showed an inhibitory effect on the growth of A549 and H1975 cells (see Figure 1C). The IC_50_ of T59 was (2.50 ± 0.58) μM in A549 and (2.91 ± 0.73) μM in H1975, but that of curcumin was much more than 10 μM. Therefore, the cytotoxicity effect of T59, compared to curcumin, in A549 and H1975 was much more effective. The cellular morphologies were detected after treatment with 0.250 μM, 0.500 μM, and 1.000 μM T59 and with 10.000 μM curcumin for 24 h, and the results show that cells gradually shrink and die as the concentration of T59 increases. The results show that T59 is more effective than curcumin to promote cell death in A549 and H1975.

### 2.2. Effects of T59 on Cell Apoptosis

**Figure 2:**
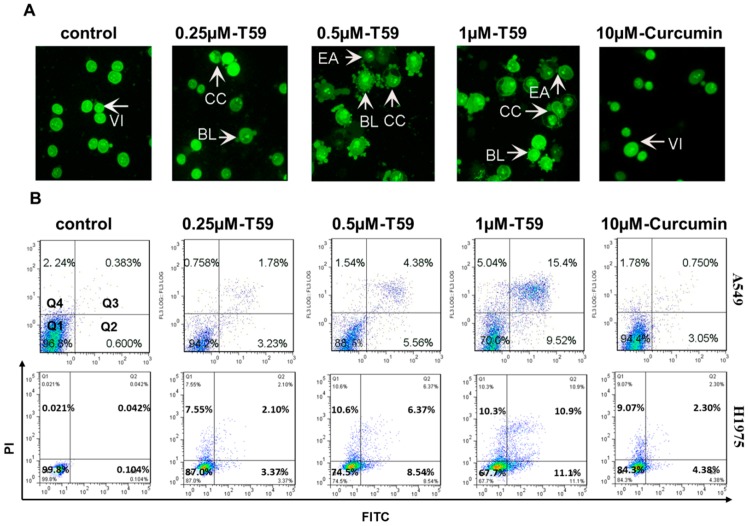

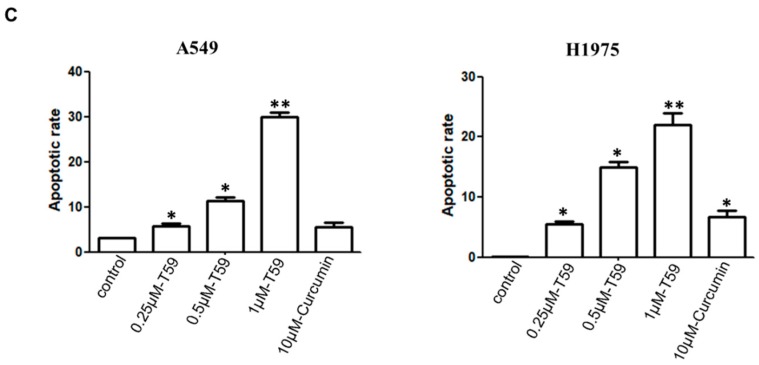
T59-induced apoptosis of A549. (**A**) Fluorescent micrographs (×400) of acridine orange (AO) stained A549 cells after treatment with 0.250 μM, 0.500 μM, and 1.000 μM T59 and with 10.000 μM curcumin for 24 h. Early apoptosis features arose after treatment with 0.500 μM T59 for 24 h. VI: viable cells; BL: blebbing of the cell membrane; CC: chromatin condensation; EA: early apoptosis. (**B**) A549 and H1975 were stained with Annexin V-FITC and PI and analyzed by flow cytometry after treatment with 0.250 μM, 0.500 μM, and 1.000 μM T59 and with 10.000 μM curcumin for 24 h. (**C**) Histogram showing the statistical apoptosis rate of the three independent experiments in (**B**). Apoptosis rate = Q2 + Q3. * *p <* 0.050 stands for statistical significance.

To prove the inhibitory nature of T59 against lung cancer cells, the cell apoptosis alteration of A549 and H1975 cells was performed by flow cytometry. After treatment with 0.250 μM, 0.500 μM, and 1.000 μM T59 and with 10.000 μM curcumin for 24 h, A549 cells were stained with acridine orange (AO) (see Figure 2A) and Annexin V-FITC/PI (see Figure 2B) and the statistical apoptosis rates were analyzed (see Figure 2C). As shown in Figure 2A, the early apoptosis features rose after cells were treated with 0.500 μM T59 for 24 h. The statistical apoptosis rates of A549 and H1975 cells were increased in a concentration-dependent manner. The percentage of apoptotic A549 cells were [(1.01 ± 0.76)%], [(5.25 ± 1.08)%], [(9.88 ± 1.49)%] and [(25.55 ± 1.21)%] when compared with the control group [(3.80 ± 1.05)%] and when treated with 0.250 μM, 0.500 μM, and 1.000 μM T59 and with 10 μM curcumin for 24 h, respectively (see Figure 2C). These results indicated that T59 caused the cell apoptosis in A549 and H1975.

### 2.3. Effects of T59 on Cell Membrane Potential

**Figure 3:**
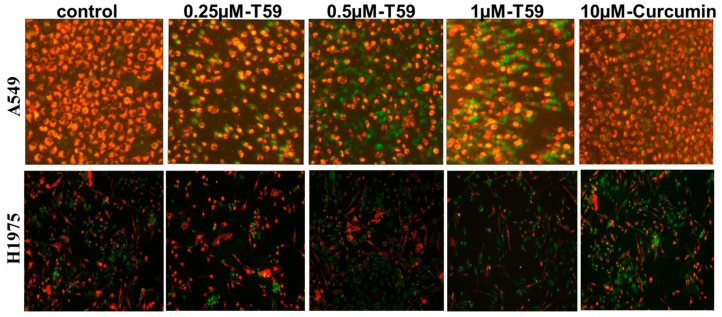
The effect of T59 on A549 cell membrane potential. A549 and H1975 cells were treated with 0.250 μM, 0.500 μM, and 1.000 μM T59 and with 10.000 μM curcumin for 24 h. Fluorescent micrographs (×100) of JC-1 stained.

The levels of mitochondrial membrane potential are given in Figure 3. The data show that the mitochondrial membrane potential levels in T59 treatment groups were significantly lower than the control and curcumin groups.

### 2.4. Effects of T59 on ROS Generation

**Figure 4:**
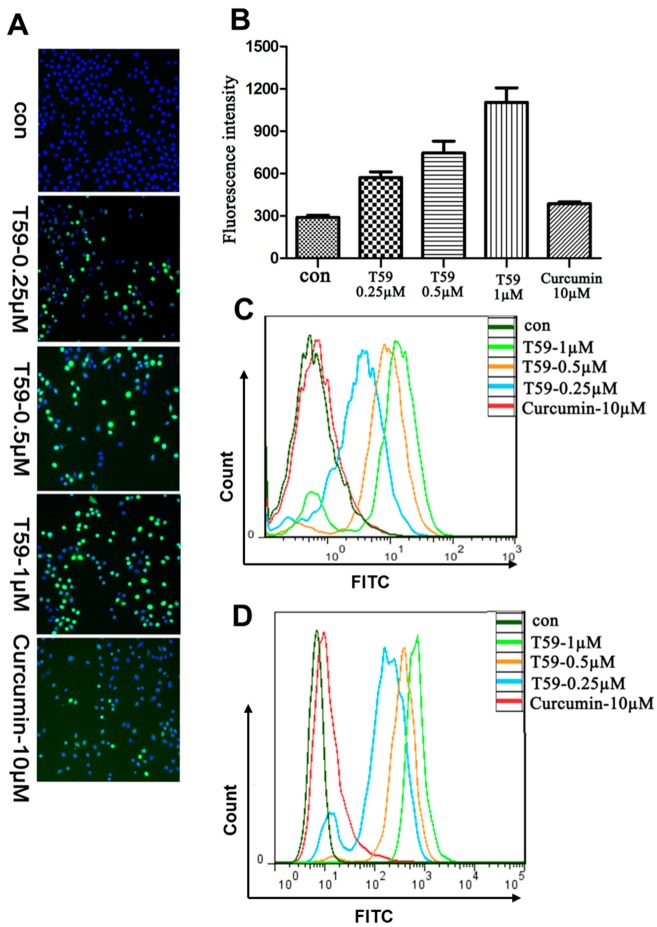
T59 induced elevated levels of ROS. (**A**) Representative images of A549 cells treated with medium including 0.250 μM, 0.500 μM, and 1.000 μM T59 and with 10.000 μM curcumin for 6 h and stained with DCFH-DA. (**B**) The fluorescence intensity in (**A**) was shown as the histogram. Data are shown as mean ± SD. (**C**) After treatment for 24 h, A549 cells were stained with DCFH-DA and analyzed by flow cytometry. (**D**) After being treated for 24 h, H1975 cells were stained with DCFH-DA and analyzed by flow cytometry.

A549 and H1975 cells were stained with DCFH-DA to determine the generation of ROS and analyzed using a fluorescence microscope and flow cytometry. The ROS levels obtained from T59 and curcumin groups are shown in Figure 4A,B. The results show that the green fluorescence was significantly increased in T59 groups when compared with the control group in a concentration-dependent manner. Moreover, the flow cytometry shows the same results (see Figure 4C,D).

### 2.5. T59 Induces Cell Apoptosis through the AKT Signaling Pathway

**Figure 5:**
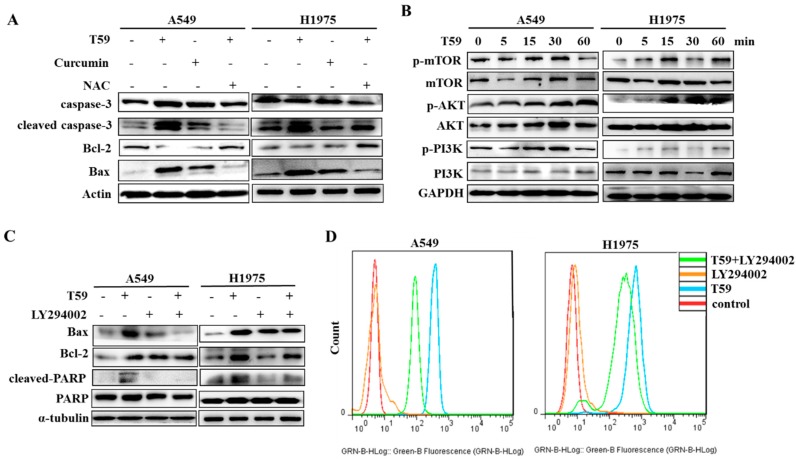
T59 induces cell apoptosis through the AKT signaling pathway. (**A**) A549 and H1975 were treated with medium of 0.500 μM T59, 10.000 μM curcumin, 0.500 μM T59 and NAC, and NAC alone. The effects of T59 on apoptosis associative proteins in A549 and H1975 were determined by the Western blot with Actin as a loading control after being treated for 24 h. (**B**) The total amount of proteins and phosphorylation proteins of mTOR, PI3K, and AKT were detected by Western blotting within 60 min of treatment of 0.500 μM T59. (**C**) Cells were treated with 0.500 μM T59 and/or LY294002 for 48 h and the expression of Bax, Bcl-2, and PCNA. Total and cleaved PARP were detected by Western blotting. (**D**) Cells were stained with DCFH-DA and analyzed by flow cytometry after treatment of 0.500 μM T59 and/or LY294002 for 24 h.

In the protein level, the damaged cells have many variations such as Bcl-2, Bax, and cleaved caspase-3 [18]. As shown in Figure 5A, the expression of cleaved caspase-3 and Bax were increased and the expression of Bcl-2 was decreased after treatment with T59, while NAC reversed that. To determine the molecular mechanisms underlying the cell apoptosis by T59, the levels of the molecules involved in the AKT signaling pathway were checked with Western blotting. The total amounts of proteins and phosphorylation proteins of mTOR, PI3K, and AKT were detected. The results show that the phosphorylation proteins of AKT in A549 and H1975 were significantly increased after stimulation by T59 (see Figure 5B), and the apoptosis effects of T59 were reversed by LY294002, which is the inhibitor of the PI3K/AKT signaling pathway (see Figure 5C). Furthermore, the generation of ROS induced by T59 was also suppressed by LY294002 (see Figure 5D).

## 3. Discussion

Curcumin, which is one of the traditional Chinese herbal formulations, exerts significant anticancer effects in many kinds of cancers. The increasing formulation of optimizations and analogues of curcumin are discovered [19,20,21]. T59 is a new formulation of optimized curcumin. However, the understanding of the role of T59 in lung cancer treatment is insufficient in current research. In order to provide more evidence of T59 in treating lung cancer cells A549 and H1975 in vitro, we analyzed the cytoxicity effects and the molecular mechanism of T59 in A549 and H1975 cells compared with curcumin.

On the one hand, the cytotoxicity of T59 was determined using the MTT assay. The results show that T59 treatment, when compared with curcumin, induced a more effective toxicity in A549 and H1975. The dose-dependent inhibition of A549 and H1975 perforation was profound with IC_50_ of [(2.50 ± 0.58)%] μM and [(2.91 ± 0.73)%] μM for 24 h. In addition, the AO staining method was used to identify the early and late phases of apoptosis in A549 cells after being treated with T59 and curcumin. Annexin V-FITC/PI was used to record the apoptotic population. Taken together, the treatment of T59 in A549 and H1975 was confirmed for apoptosis in a dose-dependent manner. On the other hand, it is universally acknowledged that the mitochondrion is the paramount control center of apoptosis [22,23,24]. As a result, the JC-1 fluorescent probe was applied to detect the changes in the mitochondrion membrane potential after being treated with T59. Our results show that T59 induced pyknosis, cell rupture, and membrane damage in A549 and H1975 cell lines. In all, these results indicated that T59 exerted cytotoxicity effects by inducing the damage of the mitochondrion membrane.

Apoptosis is the tightly controlled pattern of cell death necessary for development in organisms and typical cell growth. Mitochondrially mediated apoptosis is accompanied by the growth of ROS and the shrinkage of mitochondrion membrane potential, which induced an increased permeability [25,26]. We suggested that the pathway of apoptosis induced by T59 was mitochondrially mediated apoptosis. We observed an increase in intracellular ROS levels. Therefore, our results indicate that T59 induced A549 and H1975 cells apoptosis through increased ROS contents in cells. Caspase-3, which is a class of cysteine proteases, is a major executioner that caspase activates during cell apoptosis [27]. The Bcl-2 family such as Bcl-2, Bax, and Bad belongs to the key mediators in an intrinsic apoptotic response [28,29]. Cell apoptosis is stimulated by Bax and inhibited by Bcl-2. AKT, which is a protein kinase involved in various biological activities, has been reported to be involved in cell apoptosis. As shown in Figure 5, T59 limited the cell cycle arrest, elicited the caspase cascade, and activated the AKT signaling pathway. On the other hand, these consequences were reversed by LY294002, which is the inhibitor of the PI3K/AKT signaling pathway. Moreover, the generation of ROS induced by T59 was also suppressed by LY294002. In the current study, we found that T59 induced A549 and H1975 apoptosis by activating ROS through the PI3K/AKT signaling pathway.

To sum up, in the present study, it was established that T59 is a potent compound that induces apoptosis in human lung cancer cells in vitro. The process of apoptosis in A549 and H1975 induced by T59 was mainly attained through mitochondrially mediated apoptosis. Intrinsically, the PI3K/AKT signaling pathway occurs. These findings provide novel insights in T59 for further investigation and study in order to be applied for treating human cancers.

## 4. Materials and Methods

### 4.1. Cell Culture

Human lung cancer cell lines A549 and H1975, which were purchased from the Type Culture Collection of the Chinese Academy of Sciences (Shanghai, China), were cultured in RPMI 1640 (GIBCO-BRL, San Francisco, CA, USA) with 10% fetal bovine serum (GIBCO-BRL), 100 U/mL penicillin, and 100 μg/mL streptomycin in a humidified atmosphere at 37 °C with 5% CO_2_.

### 4.2. Cellular Viability Assay

The cells (5 × 10^4^/mL) were seeded in 96-well plates and treated with medium as a control. T59 and curcumin at concentrations of 0.078 μM, 0.156 μM, 0.313 μM, 0.625 μM, 1.250 μM, 2.500 μM, 5.000 μM, and 10.000 μM. After 24 h of treatment, 0.250 mg/mL MTT (Merck Millipore EA, Darmstadt, Germany) was added into each well and incubated for 4 h. The supernatant was removed and 100 μL of DMSO was added to each well. The absorbance at 490 nm was measured by the microplate reader (Spectra MAX 340; Molecular Devices, Sunnyvale, CA, USA).

### 4.3. Morphological Assessment of Apoptotic Cells by Acridine Orange (AO) Staining

A549 and H1975 (3 × 10^5^/mL) were plated in 12-well plates and treated with 0.250 μM, 0.500 μM, and 1.000 μM T59 and with 10.000 μM curcumin for 24 h. The supernatant was discarded and cells were washed by PBS. Ten microliters of 30 mg/L AO (Beyotime Biotechnology, Jiangsu, China) were added into the plates, and the fluorescence were observed under the UV-fluorescence microscope (NIKON Eclipse 55i, South Hadley, MA, USA).

### 4.4. Annexin V-FITC/PI Assay

After 24 h of treatment, the cells were collected and washed twice with PBS. The supernatant was removed and cells were re-suspended by the binding buffer. Five microliters of Annexin V-FITC (Wanleibio, Shenyang, China) and 10 μL of PI were added, and cells were incubated at room temperature for half an hour in the dark. The fluorescence was determined by flow cytometric analysis (Beckman-Coulter, Miami, FL, USA).

### 4.5. JC-1 Dye for Mitochondrial Membrane Potential

Mitochondrial membrane potential was measured by fluorescence tetraethyl benzimidazoly carbocyanineiodide (JC-1) assay kit (Abcam, Cambridge, UK). A549 and H1975 cells were seeded in 12-well plates. After treatment for 24 h, cells were exposed to the JC-1 dye (1 μM) and imaged using the fluorescent microscope.

### 4.6. Reactive Oxygen Species Assay

Treated for 24 h, cells were incubated in free FBS medium with 10 μM DCFH-DA (Beyotime Biotechnology, Jiangsu, China) for 30 min. High Content Analysis (HCA) was used to image the fluorescence and flow cytometry was used to analyze the fluorescence value.

### 4.7. Western Blotting

A549 and H1975 cell lines were seeded into six-well plates. Following treatment, cells were lysed in RIPA at ice cold temperatures. The supernatants were conserved after being centrifuged at 12,000 rpm for 20 min. The concentrations of total protein were determined by using the Bradford assay (Bio-Rad Laboratories, Hercules, CA, USA). The equivalent protein was electrophoresed on the sodium dodecyl sulfate polyacrylamide gel (SDS-PAGE) and then transferred onto polyvinilydenedifouride (PVDF) membrane (Millipore, Billerica, MA, USA). The membrane was blocked with 5% nonfat dried milk for 2 h at room temperature and then probed with 1:1000 dilution of primary antibody (Santa Cruz Biotechnology, Santa Cruz, CA, USA) overnight at 4 °C. After being washed by PBST three times for 30 min, the membranes were incubated in a horseradish peroxidase (HRP) conjugated secondary antibody (1:5000 in 5% nonfat dried milk) for 2 h at room temperature. The proteins were visualized by chemiluminescence (ECL kit, Beyotime Biotechnology, Jiangsu, China) in Ta anon 5200 Multi instrument.

### 4.8. Statistical Analysis

Three similar experiments were performed. The data was expressed as a mean ± standard deviation (SD). A Student’s *t*-test was used to determine the significant differences between different groups. * *p <* 0.050 stands for statistical significance. ** *p <* 0.010 means high statistical significance.
